# A pathogenic haplotype, common in Europeans, causes autosomal recessive albinism and uncovers missing heritability in OCA1

**DOI:** 10.1038/s41598-018-37272-5

**Published:** 2019-01-24

**Authors:** Karen Grønskov, Cathrine Jespersgaard, Gitte Hoffmann Bruun, Pernille Harris, Karen Brøndum-Nielsen, Brage S. Andresen, Thomas Rosenberg

**Affiliations:** 1 Kennedy Center, Department of Clinical Genetics, Copenhagen University Hospital, Rigshospitalet, Denmark; 2 Department of Biochemistry and Molecular Biology and the Villum Center for Bioanalytical Sciences, University of Southern Denmark, Odense, Denmark; 3 Department of Chemistry, Technical University of Denmark, Kgs. Lyngby, Denmark; 4 Department of Ophthalmology, Kennedy Center, Copenhagen University Hospital, Rigshospitalet, Glostrup Denmark

## Abstract

Oculocutaneous albinism (OCA) is a genetically heterogeneous disorder. Six genes are associated with autosomal recessive OCA (*TYR*, *OCA2*, *TYRP1*, *SLC45A2*, *SLC24A5* and *LRMDA*), and one gene, *GPR143*, is associated with X-linked ocular albinism (OA). Molecular genetic analysis provides a genetic diagnosis in approximately 60% of individuals with clinical OA/OCA. A considerably number of the remaining 40% are heterozygous for a causative sequence variation in *TYR*. To identify missing causative sequence variants in these, we used a NGS based approach, genotyping and segregation analysis. We report two putative pathogenic haplotypes which only differ by two extremely rare SNVs, indicating that the haplotypes have a common derivation. Both haplotypes segregate consistent with an autosomal recessive inheritance pattern and include the allele p.S192Y-p.R402Q. An explanation for the pathogenicity of the haplotypes could be the combination of p.S192Y and p.R402Q. Homozygosity for the pathogenic haplotypes causes a partial albinism phenotype. In our cohort, 15% of affected individuals had a molecular genetic diagnosis involving the pathogenic haplotype. Consequently, the prevalence of albinism seems to be substantially underestimated, and children with unexplained bilateral subnormal vision and/or nystagmus should be analysed clinically and molecularly for albinism.

## Introduction

Oculocutaneous albinism (OCA) is a genetically heterogeneous disorder which affects eyes, skin and hair. Six genes are known to be associated with OCA: *TYR* (OCA1), *OCA2* (OCA2), *TYRP1* (OCA3), *SLC45A2* (OCA4), *SLC24A5* (OCA6) and *LRMDA* (OCA7) which all show autosomal recessive inheritance^1^. Ocular albinism (OA) which only affects the eyes can be caused by mutations in *GPR143*, which is located on the X-chromosome. Autosomal recessive OA (AROA) has been ascribed to either hypomorphic mutations in genes already known to be associated with OCA or mutations in genes not yet known to be associated with albinism. For all types of albinism, the ocular signs are nystagmus, refractive errors, reduced visual acuity, iris hypoplasia, peripheral retinal pallor, foveal hypoplasia, and misrouting of the optic pathways. None of these signs, however, are either obligate or diagnostic^2^.

Molecular genetic analysis of the seven genes listed above, provides a genetic diagnosis in approximately half of the cases. This leaves relatively many affected individuals without a genetic diagnosis and an unexpectedly large proportion of these are heterozygous for mutations in *TYR*. With the implementation of Next Generation Sequencing (NGS), it has become possible to investigate non-coding regions of the genes and perform copy number variation (CNV) analysis.

## Results

### NGS analysis

An overview of the workflow is depicted in Fig. S1. To search for the missing mutations, the genomic region of *TYR* was sequenced in 15 individuals with albinism of whom 12 were heterozygous for a mutation in *TYR* and three were heterozygous for a mutation in both *TYR* and *OCA2* (Fig. S1 box 2; Table S1). Data analysis revealed a rare SNV (rs147546939A/G, c.1185-6208A > G) located in intron 3, of which the minor allele (G) were present in 11 out of the 15 individuals (Fig. S1 box 3). We further genotyped 78 individuals for rs147546939 (Fig. S1 box 4). In total, out of 93 individuals with albinism, 26 were heterozygous and six were homozygous for rs147546939G (38/186 alleles, MAF: 0.2043) (Fig. S1 box 3 and 5). This is significantly increased compared to both a control group from the Genome Denmark study^3^ where rs147546939G was found in five out of 300 alleles (MAF = 0.0167) (P < 0.0001) and in the gnomAD database (version r2.0.2) where rs147546939G is reported in 269 out of 14956 alleles (non-Finnish Europeans, MAF = 0.018) (Tables 1; S1). Noteworthy, rs147546939G is exclusively present in individuals with albinism who are either heterozygous for a *TYR* variant (21/58 alleles, MAF = 0.362) or mutation negative (17/94 alleles, MAF = 0.1809) (Fig. S1 box 3 and 5). Therefore, rs147546939G is substantially enriched in individuals with albinism and specifically in those with only one identified pathogenic variant in *TYR* (P < 0.0001). rs147546939G was not found in any of 32 unaffected family members shown to be carriers of a pathogenic *TYR* variant.

**Table 1:** MAFs rs147546939.

SNP	Chr. Position (chr 11)	Alleles	MAFa	MAFb	OCA cohort	TYR het and no mutation
rs187887338	88811249	C/G	0.01130	0.0167	NA	NA
rs145409367	88845187	C/T	0.008135	0.0067	NA	NA
rs1042602 (p.Ser192Tyr)	88911696	C/A (S/Y)	0.3640	0.31	0.5783	0.7326
rs529135220	88978983	G/C	0.0016	0.0067	NA	NA
rs147546939	89011733	A/G	0.01799	0.0167	0.2043	0.2533
rs1126809 (p.Arg402Gln)	89017961	G/A (R/Q)	0.2725	0.28	0.3604	0.4811

(a) Non-Finish Europeans (gnomAD database). (b) Danish ancestry (Genome Denmark study).

CNV analysis using the NGS data showed no rare deletions or duplications in the region.

### Segregation analysis

**Figure 1:**
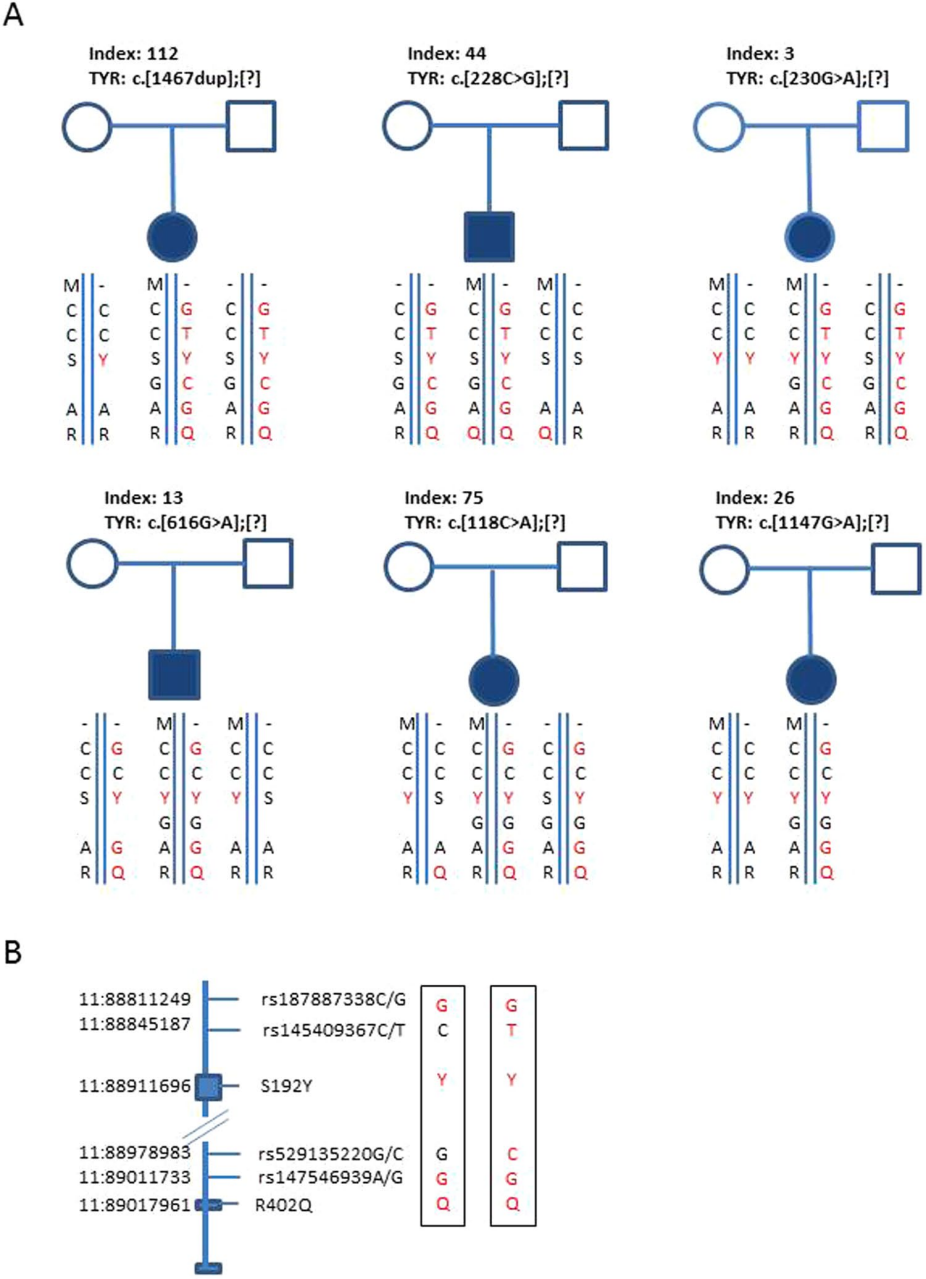
(**A**) Pedigrees for six families. Haplotypes are shown below each individual. Square indicates male, circle indicates female. Filled symbols indicate an individual affected with albinism. M: pathogenic sequence variant previously identified in *TYR*. For each family the specific change are shown above the pedigree. For clarity, M is placed on top of the haplotype, but this is not necessarily the actual position. (**B**) Selected SNVs in the haplotype. Chromosomal position are shown on the left, boxes are exons 1, 4 and 5 of *TYR*, the rs number are shown on the right, and on the far right, the two pathogenic haplotypes.

Six families were analyzed by segregation analysis, and rs147546939G was *in trans* with a pathogenic *TYR* variant in all six families (Fig. 1A). Results from the segregation analysis combined with whole genome sequencing (WGS) of nine OCA individuals (six heterozygous and three homozygous for the G allele of rs147546939) (Fig. S1 box 7) showed a common haplotype of 143 SNVs located in chr11:88811249-89057348 (hg19) (Tables 2 and S2). The haplotype included two rare SNVs (frequency below 2%): rs187887338G, located 100 kb upstream from the *TYR* translation start site, and the already described rs147546939G. A subset of individuals had further two very rare SNVs (rs145409367T and rs529155220C), which probably arose later in evolution thus resulting in two haplotypes only differing for these two SNVs (Fig. 1; Table S2). We will disregard these two SNVs. Two common coding SNVs: rs1042602A p.S192Y (192Y) and rs1126809A p.R402Q (402Q) were the only coding SNV’s in the haplotype. Rare or coding SNVs in the haplotype therefore are: rs187887338G, rs1042602A (192Y), rs147546939G and rs1126809A (402Q); the haplotype is designated: “GYGQ”.

**Table 2:** WGS of nine individuals. Haplotypes and clinical diagnosis.

ID	rs1878	rs1454	S192Y	rs5291	rs1475	R402Q	TYR mutation	Diagnosis
22	G/G	C/C	A/A (Y/Y)	G/G	G/G	A/A (Q/Q)	No mutation	AROA
96	G/G	C/T	A/A (Y/Y)	G/C	G/G	A/A (Q/Q)	No mutation	AROA
308	G/G	C/T	A/A (Y/Y)	G/C	G/G	A/A (Q/Q)	No mutation	AROA
288	C/G	C/C	C/A (S/Y)	G/G	A/G	A/A (Q/Q)	c.1075C > T	OCA
44	C/G	C/T	C/A (S/Y)	G/C	A/G	A/A (Q/Q)	c.228C > G	OCA
284	C/G	C/C	C/A (S/Y)	G/G	A/G	G/A (R/Q)	c.1467dupT	OCA
112	C/G	C/T	C/A (S/Y)	G/C	A/G	G/A (R/Q)	c.1467dupT	OCA
236	C/G	C/T	A/A (Y/Y)	G/C	A/G	G/A (R/Q)	c.915C > A	AROA
271	C/C	C/C	C/A (S/Y)	G/G	A/A	G/G (R/R)	c.915C > A	AROA

Y: tyrosine, S: serine, Q: glutamine, R: arginine, OCA: oculocutaneous albinism, AROA: autosomal recessive ocular albinism.rs1878: rs187887338, rs1454: rs145409367, S192Y: rs1042602, rs5291: rs529135220, rs1475: rs147546939, R402Q: rs1126809.

### Functional studies of rs147546939

**Figure 2:**
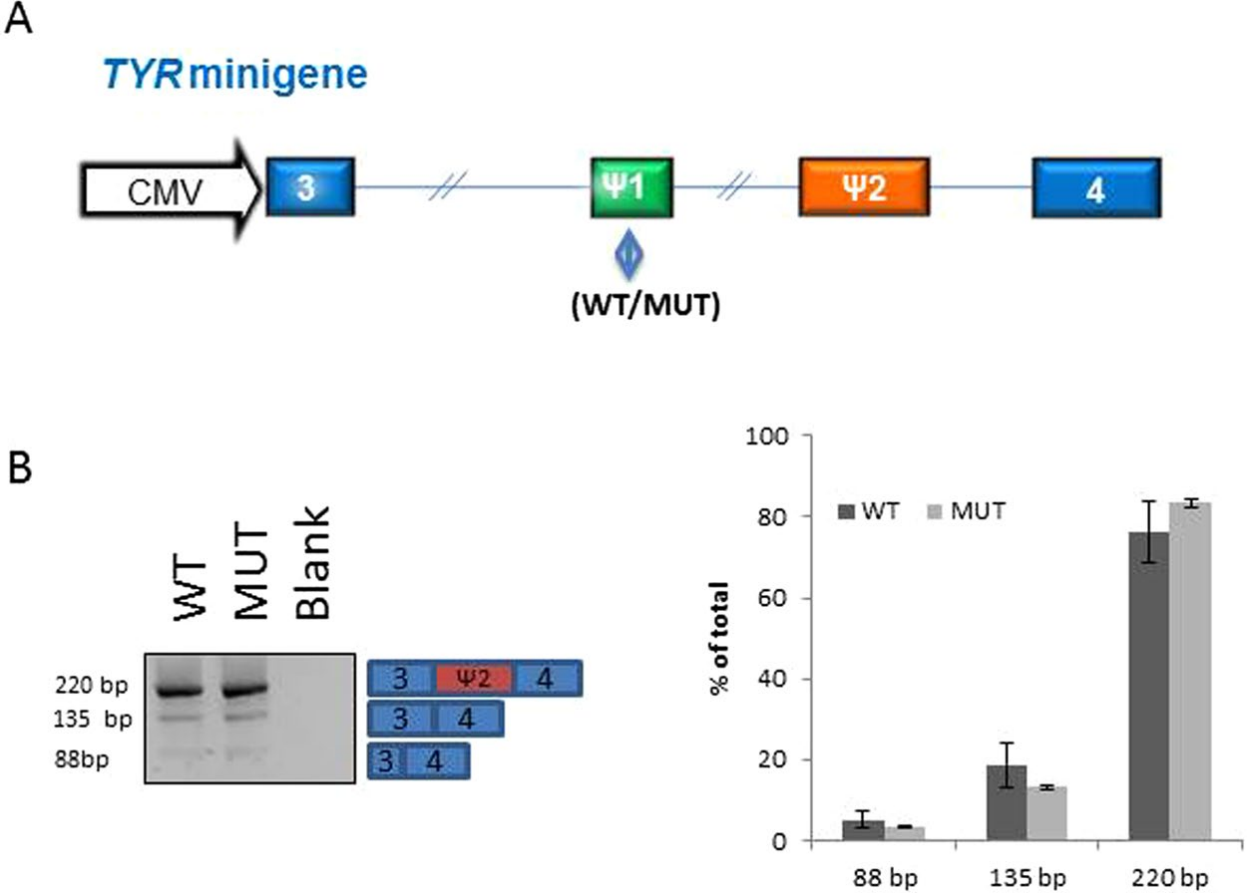
Minigene analysis of rs147546939. (**A**) The construct used for transfection. The blue boxes are exon sequences of TYR; 3: 91 bp of exon 3; 4: 47 bp of exon 4. The green box indicates the position of rs147546939, WT: rs147546939A and MUT: rs147546939G. The orange box indicates the pseudoexon. (**B**) Transfection of constructs in ARPE cells. Both constructs show inclusion of the 79 bp pseudoexon located several kb downstream of rs147546939 in the normal endogenous TYR. The identity of the bands was investigated by sequence analysis.

These results indicate that one or more of the variants shared on the haplotypes are pathogenic. Since rs147546939 is located in intron 3 of *TYR* (c.1185-6208A > G) and deep intron variants are known to be potentially pathogenic, this variant was further investigated. Analysis of the sequence surrounding rs147546939 showed two potential pseudoexons of 25 bp and 104 bp located immediately downstream of the SNV. To investigate if rs147546939G affects splicing and causes inclusion of the pseudoexon, a minigene was constructed. However, due to the large size of intron 3 of *TYR* (56802 bp) it was not possible to make a construct encompassing the full intron, instead, a construct with 1902 bp of intron 3 either with rs147546939G or rs147546939A, flanked by part of exon 3 and part of exon 4, was analysed in a minigene construct transfected in the TYR expression cell line ARPE-19^4^. Neither the minigene with rs147546939A nor that with rs147546939G included the potential pseudoexon (Fig. 2).

### Protein modeling

Protein modeling of TYR has previously been proven useful by others^5^. We therefore carried out 3D structure modeling of TYR with 402Q and with 192Y-402Q. This shows that the R402 is positioned on the surface of the protein in close vicinity of R403, H404, R405, which means that the surface charge here is highly positive. The effect of changing the arginine to a glutamine (R402Q) is illustrated Fig. S2A where the two top figures represent the wild type (R402), and the two figures below are 402Q. The red circles indicate how changing an arginine to a glutamine makes the surface less positive. It is likely that this highly positive patch is meant to bind to phosphate, sulfate or other negatively charged groups and that the change to the neutral amino acid, glutamine, will affect the ability to do so.

S192 is also positioned on the surface but opposite R402 (Fig. S2B). S192 is positioned in the loops that form the entrance of the active site. It is not unlikely that changing the serine with the more bulky tyrosine will hamper the entrance to the active site and thereby reduce the activity of the tyrosinase. Indeed the 192Y allele has been shown to have 60% of wild type activity^6^.

## Discussion

Many studies of OCA and OA have revealed that a substantial number of cases remain genetically unsolved. A considerable number of these are found to be heterozygous for a pathogenic variant in *TYR*, and the number is in excess of that explained by carrier frequency. One variant, 402Q has been subject to extensive discussion over the years, and it has been suggested to be pathogenic both on its own as well as when located in cis with 192Y^7–11^. The frequency of 28% in the gnomAD database (non-Finnish Europeans) and the identification of unaffected individuals compound heterozygous for a pathogenic variant in *TYR* and 402Q has excluded that this variant on its own is pathogenic. The frequency of 402Q in our cohort was increased in the *TYR* heterozygous and no mutation group (MAF = 0.4811 compared to genome Denmark 0.28) (P < 0.0013). An increased frequency was also observed for 192Y which has a MAF of 0.31 in the genome Denmark group compared to 0.7326 in the *TYR* heterozygous and no mutation group (P < 0.00001). Thus, even though the frequencies of 402Q and 192Y are increased in individuals with a clinical diagnosis of albinism who are heterozygous for a pathogenic variant in *TYR* or whom have no identified mutation, the frequencies are far too high to be pathogenic. However, a carrier frequency of 1.9% of the 192Y-402Q allele in a population of European ancestry has been reported^7^, and even though this frequency also seems high, we suggest that the 192Y-402Q allele is pathogenic (discussed below).

We present a pathogenic haplotype, in which pathogenic variant(s) are expected to be found among the shared variants, which are either rare or coding (GYGQ). The haplotypes segregate according to an autosomal recessive inheritance pattern and furthermore, have not been observed in unaffected carriers of a *TYR* mutation. Since the rare alleles of rs145409367 and rs529155220 are not present in all individuals these probably have arisen later in evolution and it is therefore unlikely that these SNPs are pathogenic. The deep intron variant rs147546939G was investigated by minigene analysis and demonstrated to have no effect. However, the drastic reduction in the size of *TYR* intron 3 in the minigene construct could affect the result, and we cannot completely exclude this variant as pathogenic. rs187887338G is located 100 kb upstream of the *TYR* translation in a region not evolutionary conserved and not known to possess enhancers. Thus, we have no data that indicate that rs187887338G or rs147546939G are pathogenic. The two coding variants, 192Y and 402Q have previously been suggested to be pathogenic when located in *cis*^7,8^. The study by Jagirdar *et al*.^7^ shows that in human melanocytes with genotype 402Q/Q the tyrosinase is retained in the endoplasmic reticulum (ER). The 192Y allele has been shown to have 60% of wild type activity^6^. It is plausible that the 192Y-402Q allele would result in a substantial amount of tyrosinase being retained in the ER due to 402Q, and further, that the tyrosinase released from the ER has only 60% of wild type activity due to 192Y. This could lower the available tyrosinase activity to a level that is pathogenic.

Comparison of individuals with two pathogenic *TYR* sequence variants with individuals who are compound heterozygous for the haplotype GYGQ and a pathogenic *TYR* sequence variant, shows a more severe phenotype in the former group (35 of the 37 had a clinical diagnosis of OCA compared to 15 out of 21 in the latter group; the remaining six has a clinical diagnosis of OA or AROA). Furthermore, the six individuals homozygous for GYGQ, are all from the no mutation group (N = 47). Five of the six individuals were clinically diagnosed with AROA, and thus had a mild albinism phenotype (Table 3). Jagirdar *et al*.^7^ found two individuals homozygous for 192Y-402Q who showed hypopigmentation, however no ophthalmological data were available. Thus it seems that homozygosity for GYGQ causes a mild albinism phenotype.

**Table 3:** Haplotypes and clinical diagnosis of six individuals with no previously identified mutations in TYR Y: tyrosine, Q: glutamine, OCA: oculocutaneous albinism, AROA: autosomal recessive ocular albinism.

Sample ID	rs1042602 (S192Y)	rs1126809 (R402Q)	rs147546939A > G	rs529135220G > C	Diagnosis
22	A/A (Y/Y)	A/A (Q/Q)	G/G	G/G	AROA
57	A/A (Y/Y)	A/A (Q/Q)	G/G	NA	AROA
70	A/A (Y/Y)	A/A (Q/Q)	G/G	NA	AROA
96	A/A (Y/Y)	A/A (Q/Q)	G/G	G/C	AROA
262	A/A (Y/Y)	A/A (Q/Q)	G/G	NA	OCA
308	A/A (Y/Y)	A/A (Q/Q)	G/G	G/C	AROA

The frequency of 192Y-402Q of 1.9% is comparable to the frequency of rs187887338G (MAF: 0.011) and rs147546939G (MAF: 0.018) in non-Finnish Europeans. This carrier frequency is surprisingly high if homozygosity for GYGQ causes an albinism phenotype. However, mild albinism might go undetected in a lightly pigmented population, as seen by the study of Sjöström *et al*.^12^ of the Swedish population, who found a prevalence of albinism of about 1% using iris translucency and asymmetric VEP as clinical criteria. This is in contrast to a study of children of Mexican origin where a prevalence of 0.3% was found^13^, and interestingly, the GYGQ haplotype is much less frequent in Latino population as indicated by the lower frequency of rs187887338G (MAF: 0.006) and rs147546939G (MAF: 0.008). We cannot completely rule out the possibility of an as yet undetected mutation linked to the GYGQ haplotype, however, WGS of nine individuals showed no other mutations including CNVs (deletions or duplications).

In future studies, it would be relevant to introduce the 192Y-402Q allele on a different haplotype background than the one described in this study, and measure the tyrosinase activity, however, this is out of scope for this study.

In conclusion, we report a pathogenic haplotype GYGQ in *TYR*, which may explain 15% of individuals with albinism in our cohort. The haplotype seems to result in a mild phenotype both when *in trans* to a pathogenic mutation in *TYR* and when homozygous. Our results indicate that the prevalence of albinism in European descendants is underestimated, and albinism should be considered as a diagnosis in children with subnormal visual acuity and/or otherwise unexplained nystagmus.

## Methods

### Subjects and clinical examinations

Ninety-three individuals were selected from a cohort of 177 individuals with a clinical diagnosis of albinism (OCA, AROA or OA) (Table S1, Fig. S1). All individuals were of Scandinavian descent. Most individuals were examined at a National, ophthalmological diagnostic and rehabilitation centre and selected on the basis of the presence of either congenital nystagmus or a visual acuity below 0.3 or both. We used semi-quantitative phenotype assessment to classify patients as either OCA or AROA. In brief, the diagnostic criteria for albinism included nystagmus, reduced visual acuity, iris translucency, fundus hypopigmentation, and foveal hypoplasia. Crossed asymmetry of the retino-cortical projections (misrouting) was used as an additional sign if present.

The project was approved by The National Committee on Health Research Ethics (Denmark). The project was performed according to the Declaration of Helsinki and approved by the Regional Ethics Committee. Written informed consent was obtained from all the participants and if under the age of 18 from a parent or legal guardian, before the molecular genetic testing.

### DNA sequence analysis

Genomic DNA was extracted from peripheral blood using standard protocols. Individuals were subject to molecular genetic analysis either by Sanger sequencing sequentially of the genes *TYR*, *OCA2*, *TYRP1*, *SLC45A2*, *LRMDA* and *GPR143* or by a NGS panel (*TYR*, *OCA2*, *TYRP1*, *SLC45A2*, *LRMDA*, *GPR143*, *AP3B1*, *BLOC1S3*, *BLOC1S6*, *DTNBP1*, *HPS1*, *HPS3*, *HPS4*, *HPS5*, *HPS6*, *LYST*, *MITF*, *MLPH*, *MYO5A*, *RAB27A*, *SLC38A8*).

Fifteen individuals were selected for targeted NGS analysis of the genomic regions of *TYR* (chr11:88900000-89031900, hg19). Targeted libraries for NGS were prepared using Haloplex capture technology (Agilent, Santa Clara, CA, USA). The enriched DNA libraries were sequenced using the MiSeq platform (Illumina, San Diego, CA, USA). The reads were processed using the SureCall program (Agilent), for alignment to the human genome (GRCh37/hg19) with Burrows-Wheeler Aligner (BWA-MEM) and variant calling (SNPPET).

To improve coverage, nine individuals were further analysed by whole genome sequencing and data analysis of the *TYR* genomic region (chr11:88811249-89057348). Genomic DNA was fragmented by an ultrasonicator, purified, blunt ended, ‘A’ tailed, and adaptor ligated. DNA templates with adapters were enriched using PCR and the concentrations of the libraries were determined. The libraries were sequenced on the Illumina HiSeq platform using paired-end reads according to Illumina manufacturer’s instructions (Illumina). The reads were processed following the Genome Analysis Toolkit (GATK) (https://www.broadinstitute.org/gatk/guide/best-practices). The reads were mapped to the human reference genome (GRCh37/hg19) with BWA, duplicate reads removed by Picard tools (https://broadinstitute.github.io/picard/), variants were called by the HaplotypeCaller of GATK 3,7,8,10.

### CNV analysis

The Copy Number Variants (CNVs) were called using the CNVnator read-depth algorithm^14^. The algorithm divides the genome into non overlapping bins of equal size and uses the count of mapped reads in each bin as the Read-Depth signal. We used standard settings and a bin size of 100 bp.

### Genotyping of rs147546939, rs529135220, rs187887338 and rs145409367

Analysis of rs147546939, rs529135220 and rs187887338 was performed by PCR analysis followed by Sanger sequencing using BigDye terminator v3.2 and analysis on an ABI3100XL platform. Primers used for PCR were as follows: rs147546939: Rs147546939-FH acc cac tgc tta ctg gct tat cAG GGC AGA AAA CTT CAC CAA and Rs147546939-RH gag ggg caa aca aca gat ggc TCA ACA TGG TTC TTT AAT GTC CAC; rs529135220: TYR-c1184-17845-FH acc cac tgc tta ctg gct tat cGG CGG ATT ATG AGG TCA GGA and TYR-c1184-7845-RH gag ggg caa aca aca gat ggc TTT GGC CCA CTG TTT GTT CC; rs187887338: TYR-s187887338-FH acc cac tgc tta ctg gct tat cTC TCG CTC ATA GGT GGG AAC and TYR-s187887338-RH gag ggg caa aca aca gat ggc CCA TGA AAT CTT TGG GGC ACA. Bases in lower case letters were used for sequencing and are not gene specific. Analysis of rs145409367 was performed by PCR using primers TYR-s145409367-FH acc cac tgc tta ctg gct tat cTG GAT TTG GCA AAG GTG TTT T and TYR-s145409367-RH gag ggg caa aca aca gat ggc TGA ATC CTA CTA ATC CAA TGG CT followed by restrictionenzyme analysis with HpyCH4IV (New England Biolabs, Ipswich, MA, USA) which only cuts the C allele (major allele).

### Minigene analysis

A wild type and a mutant *TYR* minigene carrying the rs147546939 mutation was constructed and cloned into pcDNA3.1 by Genscript. The minigene contains *TYR* exon 3 (91 bp), 1902 bp of intron 3 and exon 4 (47 bp). Due to its large size, intron 3 was size-reduced. The minigenes were transfected in duplicates into ARPE-19 cells, which are known to express *TYR*. RNA was isolated and used as template for cDNA synthesis. The splicing of *TYR* intron 3 was examined by PCR using primers TYR1S AAC TTA AGC TTG GTA CCT TGC ACA TC and TYR2AS GCC TTC GGA GCC ACT GCT C and subsequently analyzed by agarose gel electrophoresis. The splicing of the *TYR* minigene was quantified using the fragment analyzer (Advanced Analytical AATI).

### Protein modeling

We modelled the three-dimensional structure of the human tyrosinase protein to predict the effect of the S192Y and the R402Q mutations on protein structure/function. The human tyrosinase related protein 1 (TYRP1) (PDB entry 5mq8_A)^15^, which has a sequence identity with human tyrosinase of 44.37% was chosen as the best template for a homology model using an automatic run of SWISS-MODEL^16^. The resulting structure was investigated using Pymol^17^.

### Statistics

Fisher’s exact test, two tailed, was performed to test for significance.

## Supplementary information

Supplementary inormation
