# Supplementary material for: A pathogenic haplotype, common in Europeans, causes autosomal recessive albinism and uncovers missing heritability in OCA1
Source: Sci Rep. 2019 Jan 24;9:645. doi: 10.1038/s41598-018-37272-5 (PMC6345944; doi:10.1038/s41598-018-37272-5)
Supplement: Supplementary file 1 — Supplementary inormation [file 41598_2018_37272_MOESM1_ESM.pdf]

A pathogenic haplotype, common in Europeans, causes autosomal recessive albinism and uncovers missing heritability in OCA1

Karen Grønskov[1,*], Cathrine Jespersgaard[1], Gitte Hoffmann Bruun[2], Pernille Harris[3], Karen Brøndum-Nielsen[1], Brage S. Andresen[2], and Thomas Rosenberg[4]

Table S1. Overview of individuals investigated by targeted NGS and genotyped for rs147546939 based on OCA genotype.

**Genotype of affected individuals**	**Number of individuals**	**Individuals with rs147546939G**
Investigated by targeted NGS		
TYR (1 mut)	12	9 (het)
TYR (1 mut)+OCA2 (1 mut)	3	2 (het)
Genotyped for rs147546939		
TYR (1 mut)	12	10 (het)
TYR (1 mut)+OCA2 (1 mut)	2	0
OCA2 (1 mut)	14	0
TYR (2 mut)	2	0
TYRP1 (2 mut)	1	0
No molecular genetic diagnosis	47	5 (het), 6 (hom)

**Genotype of unaffected individuals**		
Genotyped for rs147546939		
TYR (1 mut)	32	0

Table S2. Common haplotype of 143 SNVs in chr11: 88811249-89057348.

Identifier	Ref/Alt	Haplotype	1kG Variant Frequencies (CAF)	Chr:Pos
**rs187887338**	C/G	G	0.9972,0.001797,0.0009984	11:88811249
**rs6482998**	A/T	T	0.07089,0.9291	11:88814687
**rs1815839**	T/C	C	0.00639,0.9936	11:88815236
**rs4121746**	T/C	C	0.001797,0.9982	11:88821720
**rs10765185**	A/G	G	0.07468,0.9253	11:88828091
**rs10830208**	T/C	C	0.5457,0.4543	11:88828356
**rs61903389**	T/A	A	0.8786,0.1214	11:88828429
**rs10765186**	A/T	T	0.5515,0.4485	11:88831337
**rs10830209**	A/G	G	0.5479,0.4521	11:88838984
**rs1942496**	G/A	A	0.5485,0.4515	11:88840925
**rs145409367**	C/T	C/T	0.9958,0.004193	11:88845187
**rs113279829**	T/C	C	0.5453,0.4547	11:88845892
**rs61903391**	C/T	T	0.2231, 07769	11:88845966
**rs10741302**	T/C	C	0.07029,0.9297	11:88854981
**rs1942485**	A/G	G	0.123,0.877	11:88856444
**rs10765188**	T/C	C	0.07648,0.9235	11:88858479
**rs4121730**	C/T	T	0.07947,0.9205	11:88860739
**rs11018488**	A/T	T	0.874,0.126	11:88861590
**rs10830220**	G/C	C	0.5783,0.4217	11:88874152
**rs7119848**	G/A	A	0.1709,0.8291	11:88874854
**rs4121745**	A/G	G	0.2049,0.7951	11:88874930
**rs4121744**	T/C	C	0.5563,0.4437	11:88874969
**rs7342208**	A/G	G	0.4714,0.5286	11:88876845
**rs10830221**	A/G	G	0.4311,0.5689	11:88877463
**rs4315072**	A/T	T	0.5429,0.4571	11:88878052
**rs1942492**	C/T	T	0.2851,0.7149	11:88878204
**rs4519092**	G/A	A	0.5317,0.4683	11:88880402
**rs7127310**	G/C	C	0.2869,0.7131	11:88881951
**rs10765190**	C/T	T	0.5561,0.4439	11:88882425
**rs10765191**	G/A	A	0.5333,0.4667	11:88882550
**rs10741304**	C/T	T	0.152,0.848	11:88882995
**rs11018510**	A/G	G	0.5078,0.4922	11:88883499
**rs11018511**	C/A	A	0.5563,0.4437	11:88883648
**rs10830223**	A/G	G	0.507,0.493	11:88883885
**rs10830224**	T/C	C	0.5004,0.4996	11:88884079
**rs10765192**	G/A	A	0.2336,0.7664	11:88884329
**rs4255542**	C/T	T	0.4994,0.5006	11:88885187
**rs4397857**	C/T	T	0.5875,0.4125	11:88885480
**rs10830225**	G/A	A	0.4736,0.5264	11:88885945
**rs4309163**	A/T	T	0.501,0.499	11:88886767

**rs11821161**	C/T	T	0.4075,0.5925	11:88888262
**rs10830226**	G/A	A	0.2119,0.7881	11:88889412
**rs12799122**	G/A	A	0.1755,0.8245	11:88889765
**rs61903411**	C/T	T	0.4287,0.5713	11:88889868
**rs35016324**	C/T	T	0.4287,0.5713	11:88889886
**rs35148124**	G/T	T	0.4287,0.5713	11:88889893
**rs61903412**	G/A	A	0.5315,0.4685	11:88890451
**rs10830227**	G/A	A	0.4321,0.5679	11:88890822
**rs4146871**	A/T	T	0.09605,0.904	11:88891023
**rs10830228**	A/G	G	0.4481,0.5519	11:88891114
**rs10830229**	A/G	G	0.1881,0.8119	11:88891267
**rs4081455**	C/T	T	0.4313,0.5687	11:88891543
**rs4998767**	T/A	A	0.4756,0.5244	11:88891876
**rs10830230**	C/T	T	0.4273,0.5727	11:88894537
**rs11018515**	A/G	G	0.4273,0.5727	11:88894768
**rs2212751**	A/G	G	0.4219,0.5781	11:88894922
**rs10830231**	C/T	T	0.4217,0.5783	11:88895388
**rs10830232**	A/T	T	0.4217,0.5783	11:88895962
**rs62858761**	A/C	C	0.4217,0.5783	11:88895963
**rs7108473**	C/T	T	0.4277,0.5723	11:88896425
**rs7127661**	T/G	G	0.472,0.528	11:88896609
**rs7124483**	A/G	G	0.4726,0.5274	11:88896664
**rs10830234**	A/G	G	0.4994,0.5006	11:88897444
**rs7947287**	C/T	T	0.4233,0.5767	11:88899362
**rs7950415**	C/T	T	0.4998,0.5002	11:88899605
**rs1942481**	G/A	A	0.4233,0.5767	11:88899728
**rs10765195**	T/C	C	0.5813,0.4187	11:88901170
**rs11018519**	G/A	A	0.5813,0.4187	11:88901570
**rs10830237**	C/T	T	0.4706,0.5294	11:88902144
**rs949538**	A/G	G	0.2021,0.7979	11:88902404
**rs949539**	A/T	T	0.4295,0.5705	11:88902724
**rs949537**	T/C	C	0.4778,0.5222	11:88902830
**rs7934747**	C/T	T	0.09645,0.9036	11:88903273
**rs1942498**	G/A	A	0.4271,0.5729	11:88903317
**rs12421727**	T/A	A	0.4271,0.5729	11:88904971
**rs12795279**	C/T	T	0.4267,0.5733	11:88905261
**rs7949891**	G/A	A	0.4335,0.5665	11:88907162
**rs7924925**	G/A	A	0.2119,0.7881	11:88907621
**rs1042602**	C/A	A	0.8766,0.1234	11:88911696
**rs535527**	A/T	T	0.0001997,0.9998	11:88928561
**rs525883**	C/T	T	0.001597,0.9984	11:88932412
**rs12285584**	C/T	T	0.8766,0.1234	11:88935088
**rs12295166**	T/C	C	0.8728,0.1272	11:88976157
**rs529135220**	G/C	G/C		11:88978983

**rs57627301**	A/G	G	0.6749,0.3251	11:88979332
**rs36055732**	A/G	G	0.6749,0.3251	11:88979334
**rs4121401**	T/C	C	0.5485,0.4515	11:88979846
**rs10741305**	T/C	C	0.368,0.632	11:88982718
**rs1892924**	C/T	T	0.0005, 0.9995	11:88984624
**rs1783963**	A/C	C	0.0005, 0.9995	11:88991191
**rs4087499**	G/C	C	0.8067,0.1933	11:89001126
**rs591260**	A/C	C	0.3734,0.6266	11:89002566
**rs645592**	T/C	C	0.373,0.627	11:89004894
**rs1847134**	A/C	C	0.8005,0.1995	11:89005253
**rs7101897**	C/T	T	0.7885,0.2115	11:89007922
**rs147546939**	A/G	G	0.9952,0.004792	11:89011733
**rs12273884**	T/C	C	0.5851,0.4149	11:89014355
**rs625401**	T/C	C	0.001398,0.9986	11:89017201
**rs1126809**	G/A	A	0.9187,0.08127	11:89017961
**rs1827430**	A/G	G	0.5851,0.4149	11:89018440
**rs598769**	C/T	T	0.4651,0.5349	11:89019737
**rs9919559**	T/C	C	0.5857,0.4143	11:89020590
**rs3900053**	T/C	C	0.7498,0.2502	11:89021065
**rs1847142**	G/A	A	0.8069,0.1931	11:89021574
**rs12363772**	G/A	A	0.8524,0.1476	11:89022446
**rs501301**	G/C	C	0.3766,0.6234	11:89022673
**rs11018562**	C/G	G	0.5865,0.4135	11:89022679
**rs4121403**	A/G	G	0.8121,0.1879	11:89024455
**rs7947262**	T/A	A	0.5867,0.4133	11:89026407
**rs10830253**	T/G	G	0.7758,0.2242	11:89028043
**rs28521275**	C/T	T	0.4044,0.5956	11:89028969
**rs4121404**	T/C	C	0.493,0.507	11:89029931
**rs7951935**	G/T	T	0.6052,0.3948	11:89030399
**rs7924538**	C/A	A	0.6164,0.3836	11:89030529
**rs1954771**	G/A	A	0.4954,0.5046	11:89033227
**rs1954772**	C/T	T	0.8077,0.1923	11:89033269
**rs11018564**	T/C	C	0.599,0.401	11:89035134
**rs3913538**	G/T	T	0.6274,0.3726	11:89035339
**rs12286459**	C/T	T	0.7829,0.2171	11:89035476
**rs1502259**	G/T	T	0.4914,0.5086	11:89036245
**rs1847140**	A/G	G	0.7829,0.2171	11:89037064
**rs1806319**	T/C	C	0.6134,0.3866	11:89037936
**rs10830254**	A/G	G	0.6092,0.3908	11:89040251
**rs11018567**	A/C	C	0.6004,0.3996	11:89042883
**rs7112446**	G/C	C	0.6052,0.3948	11:89043474
**rs7129117**	A/G	G	0.4854,0.5146	11:89043557
**rs7129131**	A/C	C	0.4856,0.5144	11:89043574
**rs3907665**	A/G	G	0.612,0.388	11:89043946

**rs10830255**	A/C	C	0.6126,0.3874	11:89044525
**rs11018569**	A/G	G	0.5964,0.4036	11:89044911
**rs655108**	T/G	G	0.3998,0.6002	11:89045079
**rs10830256**	G/T	T	0.4918,0.5082	11:89045556
**rs34993346**	A/G	G	0.7883,0.2117	11:89046097
**rs140758620**	G/C	C	0.7923,0.2077	11:89051769
**rs11018572**	C/T	T	0.6242,0.3758	11:89051880
**rs10830259**	G/C	C	0.624,0.376	11:89052162
**rs12576761**	C/G	G	0.7929,0.2071	11:89052255
**rs7950615**	G/A	A	0.7931,0.2069	11:89053302
**rs7943603**	T/C	C	0.6062,0.3938	11:89053387
**rs1967203**	A/G	G	0.4886,0.5114	11:89055293
**rs7101579**	A/G	G	0.6028,0.3972	11:89056614
**rs3750923**	T/G	G	0.6076,0.3924	11:89057130
**rs3829229**	G/A	A	0.8077,0.1923	11:89057348

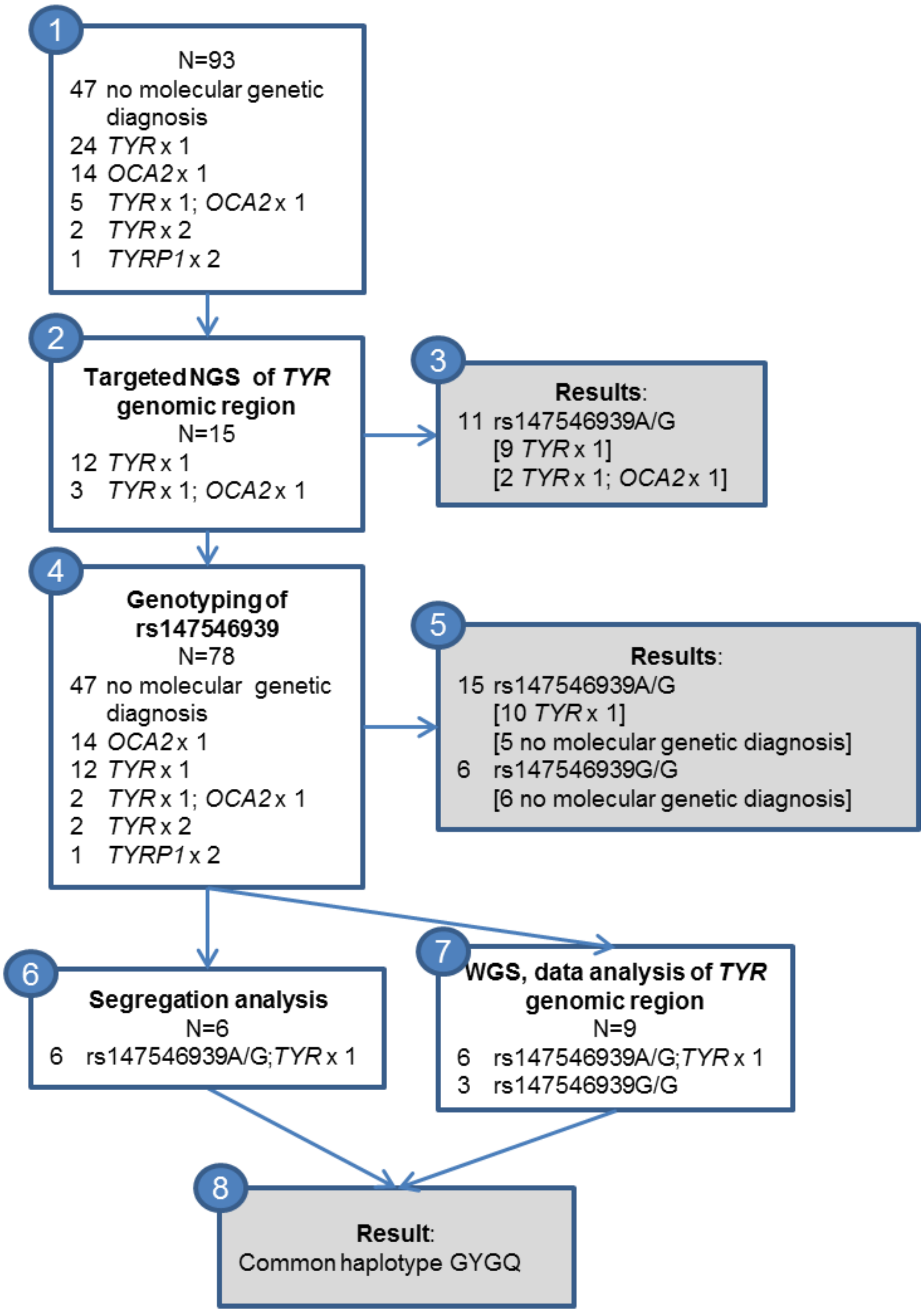

Figure S1. An overview of the workflow. Number of individuals is indicated in the boxes as N=. Below the total number is shown the number of individuals with their genotype (with respect to genes associated with OCA). Grey shaded boxes indicate a result. The number in the left upper corner of the boxes is referred to in the text.

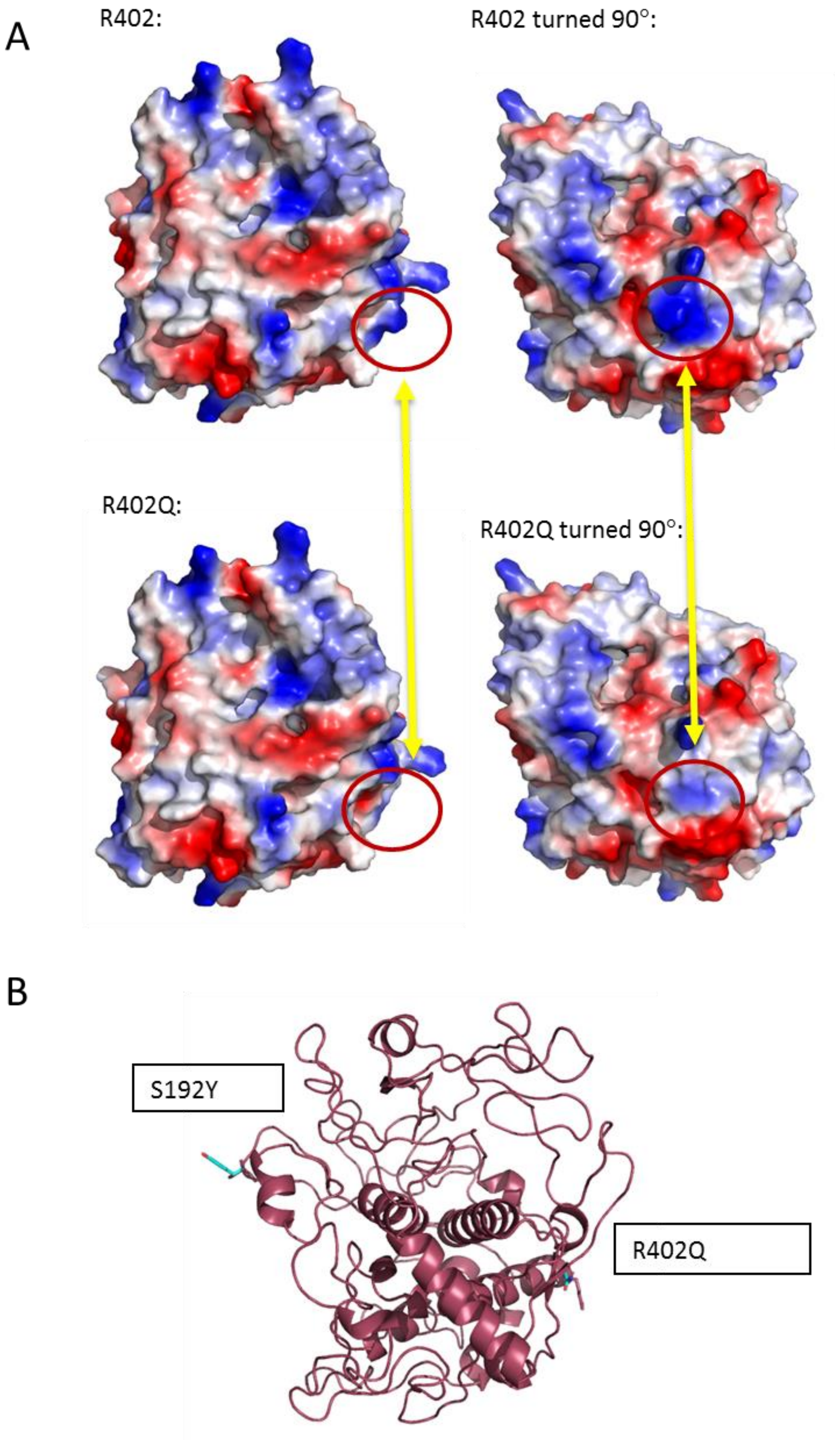

Figure S2. Protein modeling of TYR with SNVs 192Y, 402Q and 192Y-402Q using TYRP1 as a template. Figure A shows the predicted effect of the 402Q on the TYR protein. Wild type protein is shown on the two top figures, while 402Q is shown on the two bottom figures. Blue color indicates positive charge while red

color indicates negative charge. Figure B shows the predicted protein modeling with 192Y and 402Q together. 192Y are located in the loops that form the entrance of the active site.

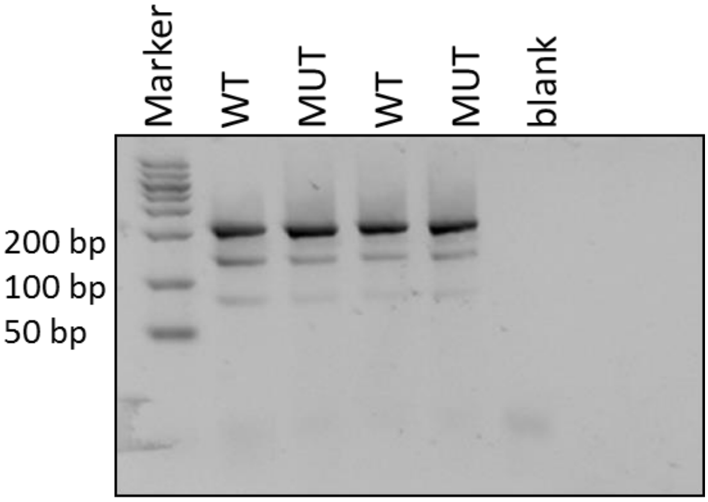

	WT	MUT
220 bp	76% ±7%	83% ±1%
135 bp	19% ±5%	13% ±1%
88 bp	5% ±2%	3% ±1%

Figure S3. Original gel picture. The cropped picture is lane 4, 5 and 6.
